# Supplementary material for: Clonal dynamics following p53 loss of heterozygosity in Kras-driven cancers
Source: Nat Commun. 2016 Sep 2;7:12685. doi: 10.1038/ncomms12685 (PMC5025814; doi:10.1038/ncomms12685)
Supplement: Supplementary Information — Supplementary Figures 1-3 and Supplementary Tables 1-2 [file ncomms12685-s1.pdf]

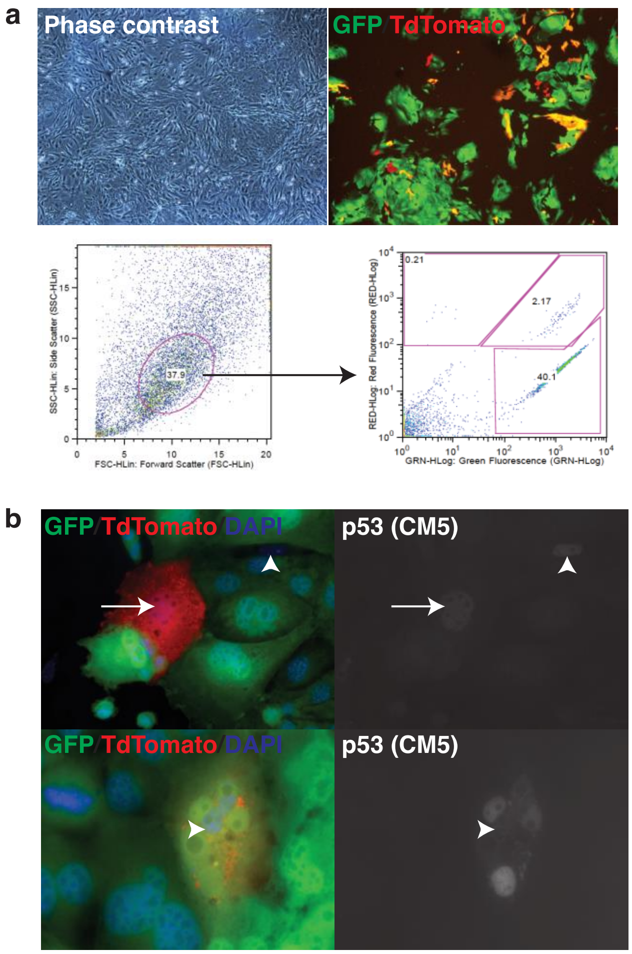

Supplementary Figure 1

Supplementary Figure 1. Induction of *p53* LOH by MADM.

a) Primary cultures derived from the pancreas of an 11-week-old *Pdx1-Cre; K-MADM-p53* mouse revealed increased $p53^{KO/KO}$ (green, GFP+/tdTomato-) cells compared to $p53^{WT/WT}$ (red, GFP-/tdTomato+) and $p53^{KO/WT}$ (yellow, GFP+/tdTomato+) cells by fluorescence microscopy and FACS. FACS plots show GFP fluorescence on x-axis and tdTomato fluorescence on y-axis.

b) Immunofluorescence staining of cells from (a) confirms nuclear p53 expression in red (arrow), yellow (horizontal arrowhead), and colorless (vertical arrowhead) cells, but not green cells.

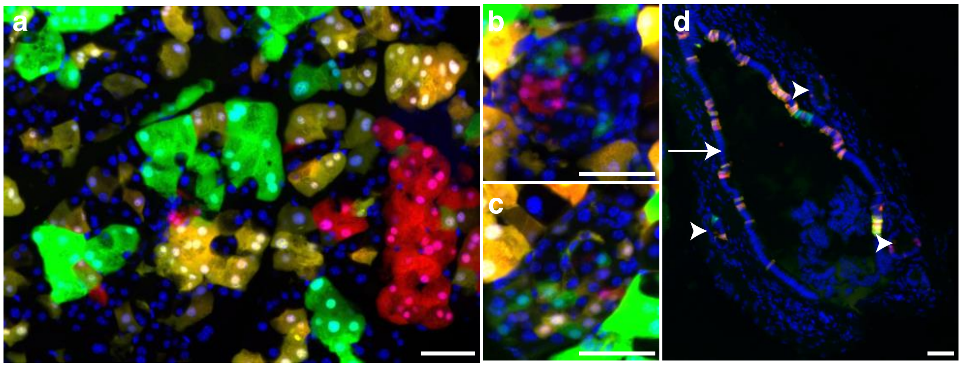

Supplementary Figure 2

Supplementary Figure 2. *Pdx1-Cre* induces MADM labeling in multiple pancreatic cell types.

a) Pancreas from a 6-month-old *Pdx1-Cre; K-MADM-p53* mouse contained $p53^{KO/KO}$ (green, GFP+/tdTomato-), $p53^{WT/WT}$ (red, GFP-/tdTomato+), and $p53^{KO/WT}$ (yellow, GFP+/tdTomato+) acinar cells with normal appearance.

b) Pancreatic islet harboring green and red cells.

c) Pancreatic islet harboring green, red, and yellow cells.

d) Large pancreatic duct (arrow) and smaller nearby ducts (arrowheads) harboring green, red, and yellow cells. Blue = DAPI-stained nuclei. All scale bars are 50 μm.

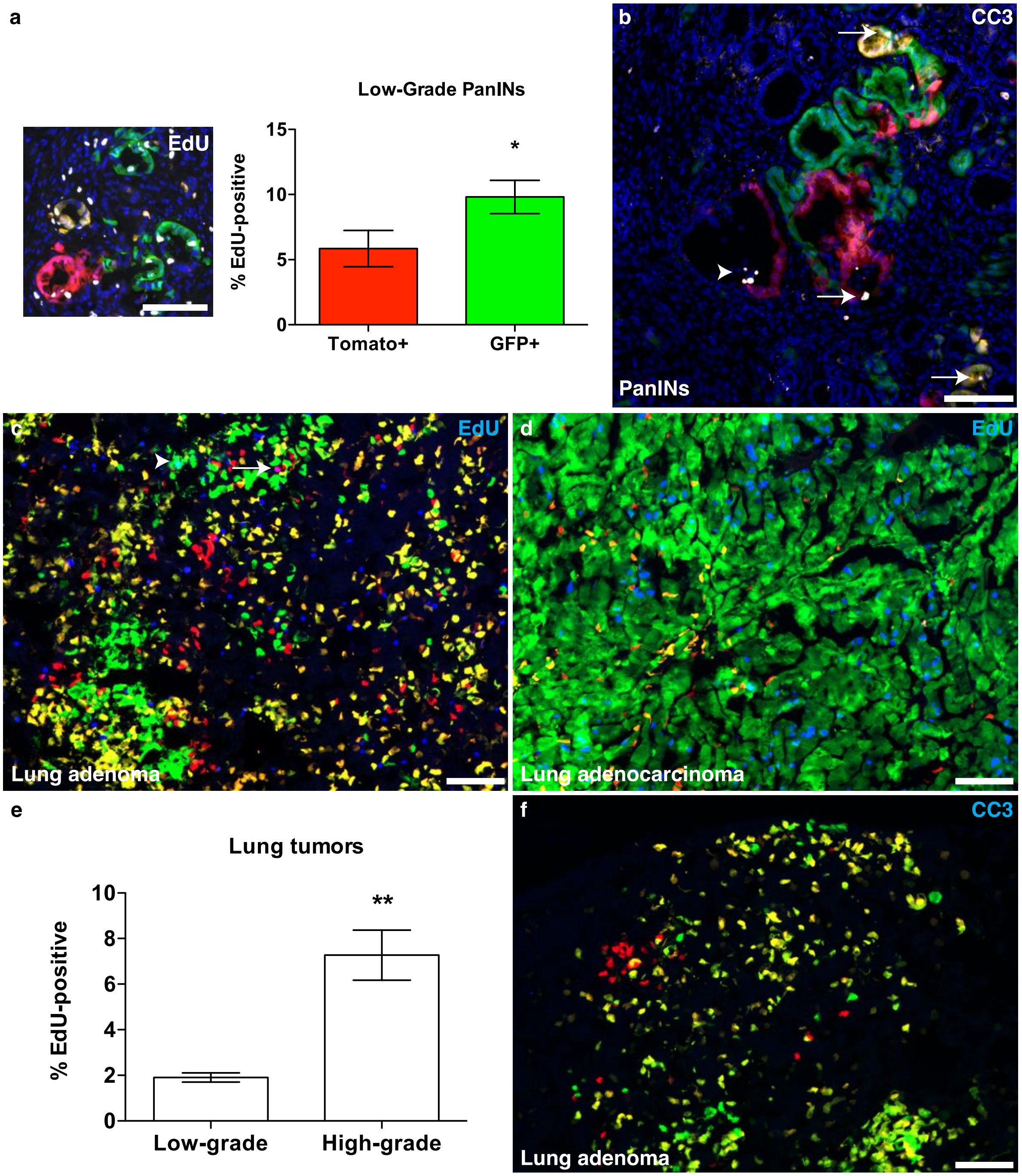

Supplementary Figure 3

Supplementary Figure 3. Analysis of proliferation and apoptosis in pancreatic and lung tumors.

a) Representative image and quantitation of EdU immunostaining (white) in low-grade PanINs from *Pdx1-Cre; K-MADM-p53* mice. Shown are the percentages of EdU-positive cells co-

labelled with Tomato or GFP (average +/- s.e.m of n=31 low-power fields from n=2 mice). *p<0.05, two-tailed student's t-test. Blue = DAPI-stained nuclei.

b) Cleaved caspase-3 (CC3) immunostaining (white) of low-grade PanINs reveals rare apoptotic $p53^{KO/KO}$ (green, GFP+/tdTomato-) $p53^{WT/WT}$ (red, GFP-/tdTomato+) and $p53^{KO/WT}$ (yellow, GFP+/tdTomato+) cells (arrows). Most apoptotic cells were found within PanIN lumens (arrowhead). No difference in apoptotic frequency based on *p53* genotype was observed. Blue = DAPI-stained nuclei.

c) EdU immunostaining (blue) of low-grade lung adenoma reveals few co-labelled $p53^{KO/KO}$ (arrowhead) and $p53^{WT/WT}$ (arrow) cells.

d) EdU immunostaining (blue) of lung adenocarcinoma reveals a many EdU-positive $p53^{KO/KO}$ cells.

e) Quantitation of the percentage of EdU-positive cells in lung tumors. Shown are average percentage +/- s.e.m. (n=28 low-grade adenomas and n=8 low-power fields of high-grade adenomas from n=3 mice). **p<0.01, two-tailed student's t-test.

f) CC3 immunostaining (blue) of low-grade lung adenoma reveals no apoptotic cells.

All scale bars are 100 μm.

Supplementary Table 1. Genotyping primers

MADM11	
Primer 1	TGG AGG AGG ACA AAC TGG TCA C
Primer 2	TTC CCT TTC TGC TTC ATC TTG C
Primer 3	TCA ATG GGC GGG GGT CGT T
Cre	
Primer 1	CAC CCT GTT ACG TAT AGC CG
Primer 2	GAG TCA TCC TTA GCG CCG TA
Primer 3	CCT TGA GGC TGT CCA AGT GAT TCA GGC CAT CG
Primer 4	CCA ATC TGC TCA CAC AGG ATA GAG AGG GCA GG
Kras^{G12D}	
Primer 1	GTC TTT CCC CAG CAC AGT GC
Primer 2	CTC TTG CCT ACG CCA CCA GCT C
Primer 3	AGC TAG CCA CCA TGG CTT GAG TAA GTC TGC A
Kras^{LA2}	
Primer 1	TGC ACA GCT TAG TGA GAC CC
Primer 2	GGA GCA AAG CTG CTA TTG GC
Primer 3	GAC TGC TCT CTT TCA CCT CC
p53$^{KO/WT}$	
Primer 1	ACC GCT ATC AGG ACA TAG CGT TGG
Primer 2	CAC AGC GTG GTG GTA CCT TAT G
Primer 3	GGT ATA CTC AGA GCC GGC CTG
p53^{flox}	
Primer 1	CAC AAA AAC AAG TTA AAC CCA G
Primer 2	AGC ACA TAG GAG GCA GAG AC
LSL-p53^{R172H}	
Primer 1	CTT GGA GAC ATA GCC ACA CTG
Primer 2	AGC TAG CCA CCA TGG CTT GAG TAA GT
Primer 3	CAA CTG TTC TAC CTC AAG AGC C

Supplementary Table 2. Genotyping protocols

Protocol	Temperature	*MADM11*	*Cre*	*Kras*^{G12D}	*Kras*^{LA2}	*p53*$^{KO/WT}$	*p53*^{flox}	*LSL-p53*^{R172H}
**Step 1**	**94C**	3:00	3:00	3:00	3:00	3:00	3:00	3:00
**Step 2**	**94C**	0:15	0:15	0:30	0:30	0:30	0:30	0:30
**Step 3**	**58C (MADM11/Cre) 60C (all others)**	0:25	0:25	1:30	1:30	1:30	1:30	1:30
**Step 4**	**72C**	0:45	0:45	1:00	1:00	1:00	1:00	1:00
**Step 2-4 Cycles**		32	32	34	34	34	34	34
**Step 5**	**72C**	5:00	5:00	5:00	5:00	5:00	5:00	5:00
**Step 6**	**4C**	forever	forever	forever	forever	forever	forever	forever
**Expected**	**WT**	350 bp	500 bp	650 bp	220 bp	450 bp	288 bp	364 bp
**Band Sizes**	**Mutant**	230 bp	300 bp	500 bp	390 bp	700 bp	370 bp	278 bp

Notes

1. Step times are listed as m:ss.
2. WT *MADM11* band corresponds to endogenous *Hipp11* locus lacking either *MADM11* allele.
3. Mutant *MADM11* band corresponds to either *MADM11-TG* and *MADM11-GT* alleles.
4. *Cre* primers are universal (detect *Pdx1-Cre* and *Rosa26-Cre*^{ERT2} transgenes).
5. WT *Cre* band corresponds to beta-globin control
